# College and Hospital Notes

**Published:** 1904-08

**Authors:** 


### COLLEGE AND HOSPITAL NOTES.

---

THE state hospital for the treatment of persons suffering from incipient tuberculosis, located at Raybrook, in the Adirondacks, was opened for the reception of patients July 1, 1904. Dr. John H. Pryor, formerly of Buffalo, is the superintendent and has been occupied for some months in making preparations for this event.

The hospital at present can accommodate about fifty patients, but there will be a camp of tents to accommodate some forty more. Only the promising cases are admitted, and those patients who can afford to pay will be required to bear the expense of their maintenance. The purpose of the state in establishing this hospital is to offer a limited opportunity for the treatment of curable cases and encourage the prevention of consumption by removing the patient from his surroundings before he becomes a source of danger.

The trustees are authorised to receive those who have no ability to pay, but no one will be admitted who has not been a citizen of the state for at least a year before his application. Any person desiring admission should apply to the poor authorities and they will arrange for the necessary examination. The county from which a free patient comes will have to pay for his care at a rate not exceeding $5 a week.

Medical examiners to determine the condition of candidates for admission to the hospital have been appointed in the larger cities. The examiners for Buffalo are Drs. H. R. Hopkins, Charles S. Jewett, B. J. Maycock, and G. T. Moseley.

---

AT THE Buffalo Hospital of the Sisters of Charity it is proposed to build a home for nurses in training at that institution. Dr. J. J. Mooney, president of the hospital staff, in his address to the graduating class of 1904, took strong ground in favor of this improvement, showing the inadequacy of the present quarters for nurses in the hospital building proper, and pointing out the further fact that the room occupied is needed for the accommodation of patients. Contributions of funds for the purpose named will be gratefully received by the governing authorities of the hospital.

---

THE Buffalo General Hospital is building a home for the nurses in the training school of that institution, which will be ready for occupancy the last week in September. In reality, it is an extension of the former nurses' home and more than doubles the present accommodations. The new building contains also a library, an assembly hall for lectures and sermons, and a gymnasium. The building stands on high ground and from an inviting roof garden a fine view of the city is obtained. When this is fitted up with awnings, hammocks, and reclining chairs, it will serve as a restful nook for tired nurses. The building will be heated with steam throughout, and will be a modern comfortable home in every respect. The cost of this improvement is estimated at $31,000.

---

A NEW quarantine hospital is building on the site of the old one on Ferry street. Dr. A. T. O'Hara, superintendent of the hospital, turned the first sod for the new building July 8, 1904.

---

THE New York School of Clinical Medicine announces the following changes in its faculty: mental diseases—Professor E. C. Dent, superintendent Manhattan State Hospital West, Ward's Island; intestinal medicine—Professor Wm. Brewster Clark, M.D.; gastrointestinal diseases—Professor Robert Coleman Kemp, M.D., Associate Professor Graham Rogers, M.D.; hydrotherapeutics—Professor Alfred W. Gardner, M.D.; ophthalmology and otology—Professor George Ash Taylor, M.D.; pediatrics—Associate Professor H. F. Senftner, M.D., Clinical Instructor and Assistant William E. West, M.D.; genitourinary diseases—Chief of Clinic and Associate Professor C. Stern, M.D.; dermatology—Chief of Clinic and Instructor L. D. Weiss, M.D. Dr. J. L. Adams was elected secretary of the faculty.

